# Seroprevalence of *Toxoplasma gondii* infection and associated risk factors among pregnant women in Debre Tabor, Northwest Ethiopia

**DOI:** 10.1186/s13104-015-1083-2

**Published:** 2015-03-29

**Authors:** Birhan Agmas, Reta Tesfaye, Digsu Negese Koye

**Affiliations:** South Gondar Zone Department of Agriculture and Rural Development, Debre Tabor, Ethiopia; Department of Veterinary Epidemiology and Public Health, Faculty of Veterinary Medicine, University of Gondar, Gondar, Ethiopia; Department of Epidemiology and Biostatistics, College of Medicine and Health Sciences, University of Gondar, Gondar, Ethiopia

**Keywords:** Seroprevalence, *Toxoplasma gondii*, Risk factors, Pregnant women, Ethiopia

## Abstract

**Background:**

*Toxoplasma gondii* is an obligate intracellular protozoan organism that infects both birds and mammals. Human infections are particularly serious if they occur during pregnancy and may result in abortion or congenitally acquired disorders which primarily affect the central nervous system. This study assessed seroprevalence of *Toxoplasma gondii* infection and associated risk factors among pregnant women at Debre Tabor, Northwest Ethiopia.

**Methods:**

An institution based cross-sectional study was conducted from February to May, 2013. A total of 263 pregnant women who came to Debre Tabor public health facilities for antenatal care were selected and included in the study. The venous blood serum was tested using toxolatex agglutination test. Data on socio-demographic and potential risk factors were collected using structured questionnaire through face-to-face interview. Data were entered and analyzed using SPSS version 20.0. Both bivariate and multivariate analyses were carried out to identify associations between dependent and independent variables.

**Results:**

Of 263 pregnant women included in the study, 180 (68.4%, 95% CI: 63.1-71.4%) were found to be seropositive for anti-*toxoplasma* antibody. Multivariable analysis showed; age group ≥36 years (Adjusted Odds Ratio [AOR] = 3.56; 95% CI: 1.01–12.5), cannot read and write (AOR = 4.77; 95% CI: 1.01-30.3), and cat ownership (AOR = 3.36; 95% CI: 1.39-8.12) were significantly associated with seropositivity of *T.gondii* infection.

**Conclusions:**

Seroprevalence of *T.gondii* infection in Debre Tabor town was high. Age, educational status and presence of cats in home were identified as factors associated with *T.gondii* infection. Education of pregnant women about the transmission and prevention methods of this infection through health extension and in antenatal care clinics is important. Besides, studies on incidence of toxoplasmosis in newborns and infants are recommended.

## Background

*Toxoplasma gondii* is an obligate intracellular protozoan organism that can cause toxoplasmosis*. T.gondii* has large number of intermediate hosts including all warm blooded animals and humans. The feline species is the definitive host for this parasite. Cat complete the coccidian life cycle with intestinal replication and passage of oocysts in the feces [1,2].

Humans are commonly infected by ingestion of raw or partly cooked meat containing *Toxoplasma* viable tissue cysts or by consumption of contaminated food and water with oocysts of *T. gondi*. It can also occur through placental transmission to the fetus [2,3].

After accidental infection of *T. gondii*, immune-competent adults and children are usually asymptomatic or cause a mild flu-like illness. Recent studies have linked toxoplasmosis with change in behavior and may be a causative or contributory factor in various psychiatric disorders such as depression, anxiety and schizophrenia [4]. However, in immune-compromised people, such as AIDS patients or pregnant women may become seriously ill, and it can occasionally be fatal. It causes severe encephalitis and neurologic diseases, and can affect the heart, liver, inner ears, and eyes (chorioretinitis). In congenitally acquired infection; a wide variety of manifestations in the fetus and infant including spontaneous abortion, still-birth, a newborn with hydrocephalus or microcephalus, cerebral calcifications and retinochoroiditis; those leads to mental retardation, blindness, epilepsy, and death. The high prevalence of *Toxoplasma* infection and its severe consequences in fetus and infant proves the importance of toxoplasmosis in pregnant women [5,6]. Antibiotic treatment to prevent fetal infection and/or fetal complications has not been very effective and the most effective way to prevent congenital Toxoplasmosis is primary prevention [7].

*T. gondii* infections during pregnancy are mostly diagnosed by detecting anti-*T. gondii-*specific IgM and IgG antibodies serologically and avidity of *T. gondii*-specific IgG antibodies. Serological tests, such as the latex agglutination (LA) test, Enzyme-linked Immunosorbent Assay (ELISA), and immunofluorescence antibody test (IFAT) are used in many clinical laboratories [6,8,9]. Latex agglutination test is relatively rapid and it does not require complex laboratory facilities. It has also high specificity (95%) and sensitivity (92%) compared to the gold standard (the Sabin-Feldman dye test). Hence it has been widely used in remote areas in developing countries [10,11].

Seroprevalence of toxoplasmosis varies from country to country. For instance, in Europe, seroprevalences of *T. gondii* infections in pregnant women vary from 9% to 67% [12]. Studies conducted in Asian countries indicated 0.8% to 28.3% seroprevalence among pregnant women [13,14]. In Africa, studies in Sudan, Burkina Faso and Cameroon showed that 25.3%, 34.1% and 70% of the pregnant women had anti-*T. gondii* antibodies respectively [15-17]. In Ethiopia, seroprevalence of 83.6% from Southwest [18] and 86.4% from central part of the country [19] were reported among pregnant women.

Studies on the prevalence of toxoplasmosis among pregnant women and associated risk factors are scanty in Ethiopia and unavailable in the study area. Therefore, this study was aimed to estimate the seroprevalence of toxoplasmosis and its associated risk factors among pregnant women attending antenatal care (ANC) at Debre Tabor town, Ethiopia.

## Methods

### Study design and area

An institution based cross-sectional study was conducted from February to May, 2013 in the three health centers and one hospital found in Debre Tabor town. Debre Tabor is 653 kilometers northwest of Addis Ababa, the capital of Ethiopia. This town is situated at latitude of 11.8500° North; longitude 38.0167° East and an altitude of 2,706 meters above sea level. It has a total population of 71,100 of which 35,372 are males and 35,728 are females [20]. Its temperature ranges from 15°C to 18.5°C.

### Study Population

The source populations were pregnant women who live in the catchment area of Debre Tabor administration. Study population comprised of all pregnant women who attended their ANC follow up at Debre Tabor public health facilities. The study participants were those pregnant women who attend ANC at these health facilities.

### Sample size and sampling technique

A single population proportion formula was used to estimate the sample size [21]. The following assumptions were made; 95% confidence level, 5% margin of error and seroprevalence of *T. gondii* among pregnant women of 83.6% from previous study in Jimma [18] and 25% for expected non-response rate. Computing with the above formula gives a total sample size of 263. About 5800 pregnant women are expected to attend ANC annually in the administration. One month data from the ANC registration book of the health institutions was taken to approximate the total number of pregnant women attending ANC during the study period and it was estimated that 966 women will attend ANC in study period. Then systematic random sampling method was used with sampling interval of 3 to select the study participants.

### Sample collection, transport and laboratory examination

Blood samples were collected from study participants who visited the health facilities for antenatal care during the study period. Three ml of venous blood was taken with plain tube by strictly following standard operational procedures. The specimens were transported to Debre Tabor General Hospital laboratory using an ice bag with ice packs. The serum was separated from the other blood constituents after clotting by centrifugation at 5000 rpm for 10 minutes. The samples were kept at −20°C till serologically tested. The serological test was done by Latex agglutination slide test. This test detects antibodies of *T.gondii* from acute (first 1–2 weeks) to lifelong previous infection. The test reagent is standardized to detect more than 10 IU/ml anti-*Toxoplasma* antibodies. The presence or absence of a visible agglutination indicates the presence or absence of anti-*Toxoplasma* antibodies in the samples tested, but it was not possible to distinguish between IgG and IgM. The test was done according to the manufacturer’s instruction (Joaquim Costa, 18, 2a planta. 08390 Montgat –Barcelona (Spain).

### Questionnaire survey

Information on socio-demographic data such as age, educational level, birth place, residence place, occupation and history of exposure for the possible associated risk factors like consumption of raw meat and vegetables, contact with cat, cat ownership, contact with soil, HIV status and other valuable information were collected by interview with structured questionnaire.

### Data management and analysis

Data obtained from both the questionnaire and laboratory tests were entered and analyzed using the statistical software SPSS version 20.0. Descriptive statistics was used to summarize the data. Variance inflation factor (VIF) was used to check multicollinearity problem. Variables which have VIF value greater than 10 are considered to be collinear variables. However, in the data set no collinearity problem was observed. Bivariate logistic regression was used primarily to check for variables that have association with the dependent variable. Variables found to have a p-value of < 0.2 in bivariate analysis were fitted into multivariable logistic regression model for controlling the possible effect of confounders and finally variables which had significant association were identified on the basis of 95% confidence interval. A p-value of less than 0.05 was taken as cut of point to declare significant association. Odds ratio was used in assessing strength of association.

### Operational definition of variables

Close contact with cat was defined as any practices that get in touch with cats or their feces such as playing with cat, clean/touch cat litter box, clean their litter and give feed for cats.

### Ethical considerations

The study protocol was reviewed and approved by the Institution Review Board of Institute of Public Health, College of Medicine and Health Sciences, University of Gondar (Ref.No SPH/1568/06/05). Official permission was also obtained from the respective bodies at Amhara Regional Health Bureau and Debre Tabor health facilities. Verbal informed consent was obtained from each study participant after being informed about the study. Participation was purely voluntary and the participants have the right to know their results. The information was kept confidentially and anonymous test was done. Treatment was given for pregnant women who were co-infected with HIV following standard protocols of the Federal Ministry of Health (FMOH) of Ethiopia [22].

## Results

### Socio-demographic characteristics of pregnant women

A total of 263 pregnant women were included in the analysis. The age range of the pregnant women studied was from 18–44 years (mean age of 29.56 years and SD ± 6.42). Seventy five (28.5%) of them attended secondary education. Majority of the pregnant women, 212 (80.6%) were urban residents and 145 (55.1%) were born at rural areas. Of the total study participants, 189 (71.9%) were house wives in occupation (Table 1).

**Table 1:** **Socio-demographic characteristics of pregnant women in Debre Tabor, Northwest; Ethiopia**

**Variables**		**Frequency**	**Percent (%)**
Age	16-20	32	12.2
	21-25	50	19.0
	26-30	67	25.5
	31-35	63	24.0
	≥36	51	19.4
Educational level	Cannot read and write	26	9.9
	Can only read and write	40	15.2
	Primary	53	20.2
	Secondary	75	28.5
	Tertiary	69	26.2
Birthplace	Urban	145	55.1
	Rural	118	44.9
Residence place	Urban	212	80.6
	Rural	51	19.4
occupation	Government employee	65	24.7
	House wife	189	71.9
	others*	9	3.4

*Merchants, private employee, students.

### Behavioral characteristics of pregnant women

Two hundred fifty one antenatal care followers had a history of exposure for one or more well-known predictor/risk factors for *Toxoplasma* infection. Sixty two (23.6%) of the pregnant women eat raw meat and 118 (44.9%) of them had cats in their home. Nearly one quarters of the pregnant women (27%) had contact with cats and garden soil (Table 2).

**Table 2:** **Behavioral characteristics of pregnant women in DebreTabor, Northwest Ethiopia**

**Variables**	**Frequency**	**Percent (%)**
**Eat raw meat**		
Yes	62	23.6
No	201	76.4
**Eat dried/cured meat**		
Yes	24	9.1
No	239	90.9
**Eat raw vegetable**		
Yes	44	16.7
No	219	83.3
**Ownership of cat**		
Yes	118	44.9
No	145	55.1
**Close contact with cat**		
Yes	71	27.0
No	192	73.0
**Source of water**		
Tap water	211	80.2
Other source*	52	19.8
**Contact with garden soil**		
Yes	71	27.0
No	192	73.0

*dug-wells, spring water sources.

### Clinical and obstetrical characteristics of pregnant women

During the time of this study, only one pregnant woman had history of blood transfusion. Two participants were found to be HIV positive; they know their HIV status during the routine HIV testing service at the ANC clinic. None of the women had history of organ transplantation. Out of 263 study participants, 102 (38.8%) were in third trimester of pregnancy. Eighty five (32.3%) of the pregnant women were nulligravidae and the remaining were multigravidae.

### Seroprevalence of *T. gondii* among pregnant women

Out of 263 samples tested, the seroprevalence of *T.gondii* was 180 (68.4%) within 95% confidence interval of 63.1%-71.4%.

### Factors associated with seropositivity

In the bivariate analysis, some variables such as age, residence, educational status, occupation, ownership of cat, close contact with cat and number of children were identified as possible risk factors associated with *T. gondii* infection. Multivariable logistic regression analysis showed that age, educational level and cat ownership were significantly associated with seropositivity of *T.gondii* infection (Table 3). Accordingly, pregnant women in the age range of 31–35 years and 36 years and above were 5.50 ( 95% CI: 1.67, 17.97) and 3.56 (95% CI: 1.01,12.51) times more likely to be seropositive respectively as compared to women in the age range of 16–20 years. Educational status of pregnant women was identified as one of the risk factors for seropositivity of *T. gondii*. Those women who cannot read and write were 4.77 (95% CI: 1.01, 30.3) times more likely to be seropositive as compared to those who attend tertiary education. Those women who had cats at their home were 3.36 (95% CI: 1.39, 8.12) times more likely to be seropositive as compared to those who didn’t have cats at their home.

**Table 3:** **Bivariate and multivariate analyses of factors associated with seroprevalnce of** ***T.gondii*** **among pregnant women, Northwest Ethiopia**

**Variables**	**Toxoplasmosis seropositivity**		**Crude OR (95% CI)**	**Adjusted OR (95% CI)**
	**Yes**	**No**		
**Age**				
16-20	16	16	1	1
21-25	25	25	0.99 (0.41, 2.43)	0.98 (0.32, 2.97)
26-30	41	26	1.58 (0.67, 3.69)	1.59 (0.55, 4.64)
31-35	54	9	6 (2.23, 16.13)*	5.50 (1.67, 17.97)*
≥36	44	7	6.29 (2.18, 18)*	3.56 (1.01, 12.51)*
**Educational level**				
Cannot read and write	24	2	5.25 (1.14, 24.27)*	4.77 (1.01, 30.3)*
Read and write only	33	7	2.06 (0.78, 5.41)	1.72 (0.58, 5.14)
Primary	45	8	2.46 (0.99, 6.11)	2.04 (0.72, 5.82)
Secondary	30	45	0.29 (0.15, 0.58)	0.40 (0.16, 0.99)
Tertiary	48	21	1	1
**Residence place**				
Urban	137	75	1	1
Rural	43	8	2.94 (1.32, 6.59)*	1.32 (0.44, 4.02)
**Occupation**				
Government employee	28	37	1	1
House wife	146	43	4.49 (2.47, 8.15)*	1.64 (0.70, 3.8)
Others	6	3	2.64 (0.61, 11.50)	0.91 (0.13, 6.54)
**Ownership of cat**				
Yes	102	16	5.48 (2.95, 10.2)*	3.36 (1.39, 8.12)*
No	78	67	1	
**Close contact with cat**				
Yes	65	6	7.25 (3, 17.6)*	2.12 (0.67, 6.67)
No	115	77	1	1
**Source of water**				
Tap water	140	71	1	
Other source	40	12	0.59 (0.29, 1.2)	-
**Contact with Soil**				
Have garden	54	17	1.66 (0.89, 3.10)	-
Have not garden	126	66	1	
**Number of children**				
No child	40	45	1	1
One child	64	20	3.60 (1.86, 6.95)*	2.22 (1.0, 4.91)
Two children	47	13	4.07 (1.93, 8.60)*	1.78 (0.67, 4.74)
Three children	22	2	12.3 (2, 55)	2.47 (0.36, 17.2)
Four or more	7	3	2.62 (0.6, 10)	0.40 (0.04, 3.72)

*Significant at P-Value <0.05.

## Discussion

In this institution based cross-sectional study, the data showed a toxo-latex agglutination test seroprevalence of 68.4% among pregnant women in Debre Tabor town, Ethiopia. This result is much higher than those reported in pregnant women from other countries like Korea 0.8%, Sudan 25.5% and Burkina Faso 34.1% [13,15,16]. It was similar to the study done in Cameroon that showed seroprevalence of 70% among pregnant women [17]. The causes for these high seroprevalence variations are attributed to environmental distinctiveness (dry climate, rainfall, temperature, soil type, altitude) and mothers’ characteristics such as management of cats, educational level, hygienic practice and feeding habit [6,23].

Former serological study in Ethiopia showed 68.8% to 92.6% prevalence in central part and 83.6% in Southwest part of the country [18,19]. The lower seroprevalence found in our study compared with those reported elsewhere might be explained by differences in altitude. The study area has an elevation of 2706 meters above sea level; high altitude does not favor oocyst sporulation and survival. Prevalence of *T. gondii* infection has been found lower in high altitudes as compared to low altitudes [24,25]. Even though it is relatively lower than other studies, 68.4% shows high burden of toxoplasmosis in the study area. This high prevalence may be due to; presence of large number of cat population and living habit of cats and humans closely, inadequate supply of water for hygienic practice. The above noted factors aid the transmission and survival of *T.gondii*. The other difference could be also due to the differences in methods used and the sensitivity difference. Two of the previous studies used ELISA which has a sensitivity of 98% [18,19].

Congenital transmission of *T. gondi* infection is a risk in areas where the disease is endemic [26]. In our finding the high prevalence obtained indicates the need for further study on incidence of this infection among infants and routine screening of women in antenatal care clinics. This study didn’t identify whether the infection is acute or not.

Age, educational level, and ownership of cat were identified as possible risk factors associated with *T. gondii* infection. The probability of having *T.gondii* antibody among pregnant women older than 36 years was 3.56 times higher than those pregnant women of 16–20 year age groups. This is in agreement with other reports from south west part of Ethiopia [18]. In dissimilarity to this finding, studies done in Thailand, Cameroon and Addis Ababa, Ethiopia, showed that seroprevalence and age were not found to have statistically significant association [14,17,27].The significant effect of age on *T.gondii* serostatus in our study subjects may suggest that the higher age group had long duration of exposure.

Women who cannot read and write were 4.77 times more likely to acquire *T. gondii* infection than those pregnant women who had tertiary level of education. This finding was in accordance with a study done in Brazil which reported pregnant women with low educational status had higher prevalence of *T.gondii* antibody [28]. Women who cannot read and write have less hygienic practice and they are more likely to be rural residents. They might have garden/agriculture and they may have close contact with animals as compared to the educated ones.

In this cross sectional study, ownership of cat was associated with seropositivity of *T.gondii*. Pregnant women who had cats at home were 3.36 times more likely to be seropositive for *T. gondii* compared with those who did not have. This finding is in agreement with reports from south west and central parts of Ethiopia, Thailand and China [14,18,19,29]. In difference; Cameroonian study found no association between sero-prevalence of *T.gondii* and ownership of cat [17]. In our study area, cats are kept mainly for the purpose of cleaning rats/mice and as pet animal. Cats are the definitive host where sexual multiplication of *T.gondii* takes place and excrete the oocysts with feces; due to this they are considered the major source of *T.gondii* infection to humans and animals [2,3].

Other behavioral risk factors such as; eating raw meat or vegetables, drinking untreated water, and contact with garden soil did not show significant association with seroprevalence of *T.gondii* in our study.

### Limitations

Further confirmatory tests/tests that can identify the type of antibody to identify acute or latent type of *T.gondii* were not done. Risk factor assessment was done based on study participants’ response which may suffer from recall bias. Since this study was institution based study, it may not represent the general population. As a cross-sectional study, it may suffer from chicken-egg dilemma.

## Conclusions

More than two thirds of the pregnant women were positive for *T.gondii* infection. This high seroprevalence of *T. gondii* infection shows the need for preventive measures such as awareness creation about toxoplasmosis; mainly education of pregnant women about the transmission and prevention methods at antenatal care clinics. Age, educational status and presence of cats at home were identified as possible associated risk factors of *T.gondii* infection. Cat owners, particularly pregnant women, should take necessary preventive measures like not eating raw meat, proper disposal of cat feces and keep hygiene to avoid *Toxoplasma* infection. Studies that show incidence of *T. gondii* among the neonates have to be done to introduce routine antenatal screening program to control congenital Toxoplasmosis.
